# Supplementary material for: T vector velocity: A new ECG biomarker for identifying drug effects on cardiac ventricular repolarization
Source: PLoS One. 2019 Jul 8;14(7):e0204712. doi: 10.1371/journal.pone.0204712 (PMC6613676; doi:10.1371/journal.pone.0204712)
Supplement: S1 Text — (PDF) [file pone.0204712.s001.pdf]

S1 Text. Rationale for choosing the Dower transformation.

Assuming that the 3-dimensional trajectory of the heart's dipole vector captures the essential information about the electrical activity of the heart measured at the body surface, we calculated this trajectory using the pseudo-inverse Dower transformation [1]. Originally, the Dower transformation was designed to linearly map Frank's lead system [2] to the standard 12 lead system. Since the ECGs of the two studies considered here were recorded using a telemetry system with Mason-Likar lead configuration, the Guldenring transformation or the Kors transformation [3] might be considered to be more appropriate. To compare the mapping performances of the various transformation options, we reconstructed the T wave signals by first applying the linear transformation from the 8 independent leads I, II, V1 to V6 onto the 3-dimensional space and then applying the pseudo-inverse transformation back to the 8-dimensional space. We quantified the reconstruction error as average root mean square (RMS) value of the residuals over the eight independent leads. Finally, the mapping performance was measured as the ratio of the residual mean RMS value over the original ECG signal mean RMS value using all ECGs of Study A. Our results showed that the Dower transformation performed much better (ratio = 0.18) than the Guldenring transformation (ratio = 0.75) and the Kors transformation (ratio = 0.61). Thus, we decided to use the Dower transformation for our analyses.

References

[1] Dower GE, Machado HB, Osborne JA. On deriving the electrocardiogram from vectoradiographic leads. Clinical Cardiology. 1980 Apr;3:87–95.

[2] Frank E. An accurate, clinically practical system for spatial vectorcardiography. Circulation. 1956 May;13:737–749.

[3] Guldenring D, Finlay DD, Strauss DG, Galeotti L, Nugent CD, Donnelly MP, et al. Transformation of the Mason-Likar 12-lead electrocardiogram to the Frank vectorcardiogram. Conference proceedings: Annual International Conference of the IEEE Engineering in Medicine and Biology
Society IEEE Engineering in Medicine and Biology Society Annual Conference. 2012;2012:677–680.
