# Supplementary material for: T vector velocity: A new ECG biomarker for identifying drug effects on cardiac ventricular repolarization
Source: PLoS One. 2019 Jul 8;14(7):e0204712. doi: 10.1371/journal.pone.0204712 (PMC6613676; doi:10.1371/journal.pone.0204712)
Supplement: S2 Text — (PDF) [file pone.0204712.s002.pdf]

S2 Text. Rationale for filter settings.

ECG signal traces are affected by different intensities and characteristics of signal noise. Since calculation of the first derivative of the signal is sensitive to the presence of high frequency noise in the raw signal, care must be taken to ensure robustness of the TVV distribution. We investigated the effect of various low-pass filter settings on the precision of the exposure response model estimates by comparing the resulting slope’s confidence intervals. We found that that using a Savitzky-Golay filter of order 3 and filter length of 31 samples produced more precise effects than similar filters with length 21 and 41.

Of note: Adjustment of the individual ECG lead signal traces to the isoelectric line is not critical for calculation of the TVV distribution, since constant offsets of the lead signals will be eliminated when calculating the first derivative of the linearly transformed ECG signal.
