# Supplementary material for: T vector velocity: A new ECG biomarker for identifying drug effects on cardiac ventricular repolarization
Source: PLoS One. 2019 Jul 8;14(7):e0204712. doi: 10.1371/journal.pone.0204712 (PMC6613676; doi:10.1371/journal.pone.0204712)
Supplement: S3 Text — (PDF) [file pone.0204712.s003.pdf]

S3 Text. Agreement of QTcF data.

In our study, ECG files were automatically processed using AbbVie's proprietary system eECG/ABBIOS. Q, J, and T_{end} annotations were visually reviewed, and manually adjusted if deemed necessary. The level of agreement of our QTcF annotations with the published QTcF data in Study A is summarized in the Bland-Altman plot (S1 Fig). On average, the published QTcF intervals data were 7.9 ms shorter than ours. Agreement was less for larger QTcF values, where the published intervals tended to be longer than ours. Given the very similar AUC values for separating potassium channel blocking drugs from multichannel blocking drugs using QTcF (0.73 versus 0.72), we conclude that differences in the Q, J, and T_{end} annotation process between our and the previously published studies have negligible impact on our current findings and reasoning.

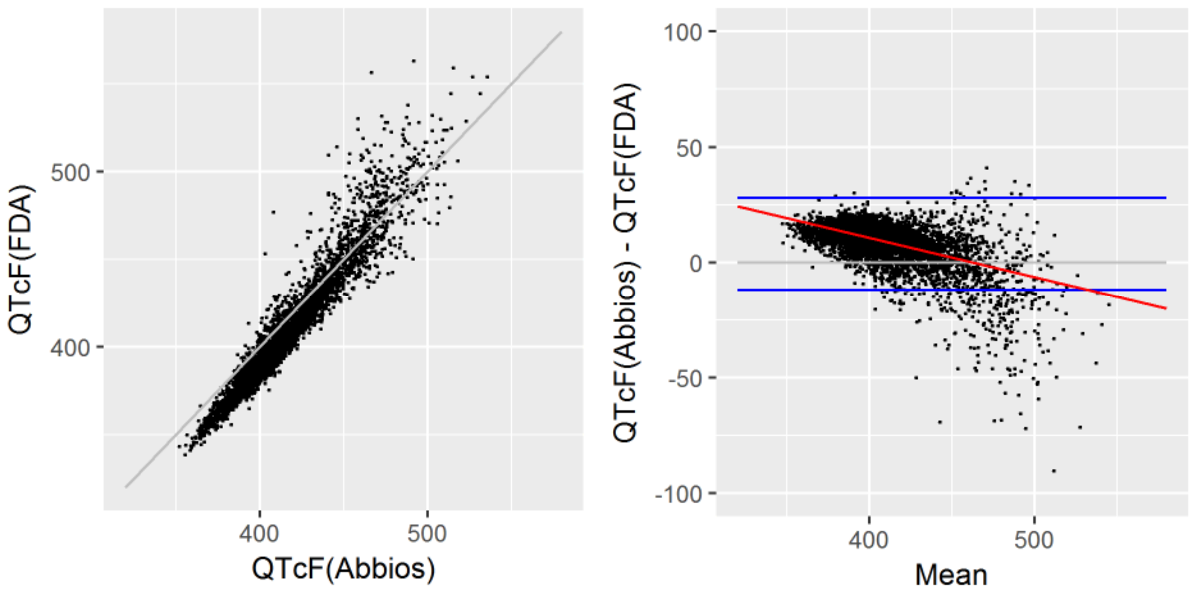

Figure 1. Comparison of QTcF between Abbios and published data from study A.
Left: Common distribution of the Fridericia corrected QT values.
Right: Bland-Altman plot of QTcF, with horizontal lines denoting the mean differences and the 95% distribution range, and the red regression line denoting the trend for the differences between shorter and longer QTcF values.
