# Supplementary material for: T vector velocity: A new ECG biomarker for identifying drug effects on cardiac ventricular repolarization
Source: PLoS One. 2019 Jul 8;14(7):e0204712. doi: 10.1371/journal.pone.0204712 (PMC6613676; doi:10.1371/journal.pone.0204712)
Supplement: S4 Text — (PDF) [file pone.0204712.s004.pdf]

S4 Text. Rationale for the heart rate correction approach.

The Tr*X* parameters denote time intervals, aligned at the J point. Thus, power-law based heart rate correction formulae like those for QTcF or J-T_{peak}c may be applicable with exponents being determined based on the drug-free data of both studies. Since the parameters presented here are a subset of a larger set of screening parameters for which power-law correction formulas were not applicable in general, we only used a basic linear regression approach for heart rate correction that was applicable identically to all investigated parameters. Reviewing the heart rate correction data, we considered the linear regression approach to be acceptable for the purposes of this study. We acknowledge that more specific heart rate correction approaches may possibly better account for the heart rate dependency of the Tr*X* parameters.
