# Supplementary material for: T vector velocity: A new ECG biomarker for identifying drug effects on cardiac ventricular repolarization
Source: PLoS One. 2019 Jul 8;14(7):e0204712. doi: 10.1371/journal.pone.0204712 (PMC6613676; doi:10.1371/journal.pone.0204712)
Supplement: S5 Text — (PDF) [file pone.0204712.s005.pdf]

S5 Text. Comparison of the drug effects between QTcF and Tr100c.

Tr100 denotes the time interval between the J point plus 20 ms and T_{end}. Thus, this parameter is equal to the QT interval minus the Q-J interval minus 20 ms. Assuming that the drugs investigated in this study have little effect on depolarization, one may expect that the drug effects reflected in the double-delta parameter values of QTcF and Tr100c are similar in size. A graphical comparison is given in Figs 5 and 6.

To statistically test equality of the double-delta changes of QTcF and Tr100c under the various treatments, we extended the mixed effects models by a pure categorial interaction term $\Psi \in \{QTcF; Tr100c\}$ for the fixed and for the random variables. For single drug treatments the mixed effects model is $\Delta\Delta P \sim 0 + \Psi{:}\,C + (0 + \Psi{:}\,C|Subject)$, and $\Delta\Delta P \sim 0 + \Psi{:}\,(C_1 * C_2) + (0 + \Psi{:}\,(C_1 * C_2)|Subject)$ for drug combinations.

We fitted the models to the pooled data from both parameters (R package lme4), and tested the equality of effects by comparing the corresponding fixed effect coefficients within a general linear hypothesis (R method multcomp:glht).

For Study A with fixed effect model coefficients β_D^{QTcF} and β_D^{Tr100c} for drug D, describing the slope of the regression line with respect to the parameter QTcF respectively Tr100c, the null hypothesis was $H_0{:}\ \beta_D^{QTcF} - \beta_D^{Tr100c} = 0$. Accordingly, for Study B with model coefficients $\beta_{D_i}^{QTcF}$, $\beta_{D_i}^{Tr100c}$, $\beta_{D_1D_2}^{QTcF}$, and $\beta_{D_1D_2}^{Tr100c}$, $i \in \{1; 2\}$, the null hypothesis for a single drug D_i was $H_0{:}\ \beta_{D_i}^{QTcF} - \beta_{D_i}^{Tr100c} = 0$. For drug combinations, the null hypothesis was $H_0{:}\ \left(M_1 * \beta_{D_1}^{QTcF} + M_2 * \beta_{D_2}^{QTcF} + M_1 * M_2 * \beta_{D_1D_2}^{QTcF}\right) - \left(M_1 * \beta_{D_1}^{Tr100c} + M_2 * \beta_{D_2}^{Tr100c} + M_1 * M_2 * \beta_{D_1D_2}^{Tr100c}\right) = 0$ with M_i as the geometric mean concentration of drug D_i determined from all timepoints with combined drug administration for both drugs D_1 and D_2.

The following table lists the p values for the various test conditions under the two-sided alternative hypothesis.

Table 1. Test for equal drug effects between QTcF and Tr100c.

Study	Treatment	Null Hypothesis	P Value, two-sided
A	Dofetilide (DOF)	$H_0\colon\ \beta_{DOF}^{QTcF} - \beta_{DOF}^{Tr100c} = 0$	0.82
	Quinidine (QUI)	$H_0\colon\ \beta_{QUI}^{QTcF} - \beta_{QUI}^{Tr100c} = 0$	< 0.001
	Ranolazine (RAN)	$H_0\colon\ \beta_{RAN}^{QTcF} - \beta_{RAN}^{Tr100c} = 0$	0.67
	Verapamil (VER)	$H_0\colon\ \beta_{VER}^{QTcF} - \beta_{VER}^{Tr100c} = 0$	0.37
B	Dofetilide (DOF)<br>+<br>Mexiletine (MEX)	$H_0\colon\ \beta_{DOF}^{QTcF} - \beta_{DOF}^{Tr100c} = 0$<br>(pure dofetilide)	0.36
		$H_0\colon\ \beta_{MEX}^{QTcF} - \beta_{MEX}^{Tr100c} = 0$<br>(pure mexiletine)	0.79
		$H_0\colon \left(M_{DOF} * \beta_{DOF}^{QTcF} + M_{MEX} * \beta_{MEX}^{QTcF} + M_{DOF} * M_{MEX} * \beta_{DOF,MEX}^{QTcF}\right) - \left(M_{DOF} * \beta_{DOF}^{Tr100c} + M_{MEX} * \beta_{MEX}^{Tr100c} + M_{DOF} * M_{MEX} * \beta_{DOF,MEX}^{Tr100c}\right) = 0$<br>(dofetilide combined with mexiletine)	< 0.01
	Dofetilide (DOF)<br>+<br>Lidocaine (LID)	$H_0\colon\ \beta_{DOF}^{QTcF} - \beta_{DOF}^{Tr100c} = 0$<br>(pure dofetilide)	0.23
		$H_0\colon\ \beta_{LID}^{QTcF} - \beta_{LID}^{Tr100c} = 0$<br>(pure lidocaine)	0.83
		$H_0\colon \left(M_{DOF} * \beta_{DOF}^{QTcF} + M_{LID} * \beta_{LID}^{QTcF} + M_{DOF} * M_{LID} * \beta_{DOF,LID}^{QTcF}\right) - \left(M_{DOF} * \beta_{DOF}^{Tr100c} + M_{LID} * \beta_{LID}^{Tr100c} + M_{DOF} * M_{LID} * \beta_{DOF,LID}^{Tr100c}\right) = 0$<br>(dofetilide combined with lidocaine)	0.10
	Moxifloxacin (MOX)<br>+<br>Diltiazem (DIL)	$H_0\colon\ \beta_{MOX}^{QTcF} - \beta_{MOX}^{Tr100c} = 0$<br>(pure moxifloxacin)	< 0.01
		$H_0\colon\ \beta_{DIL}^{QTcF} - \beta_{DIL}^{Tr100c} = 0$<br>(pure diltiazem)	0.86
		$H_0\colon \left(M_{MOX} * \beta_{MOX}^{QTcF} + M_{DIL} * \beta_{DIL}^{QTcF}\right) - \left(M_{MOX} * \beta_{MOX}^{Tr100c} + M_{DIL} * \beta_{DIL}^{Tr100c}\right) = 0$<br>(moxifloxacin combined with diltiazem,<br>model without interaction between moxifloxacin and diltiazem concentrations)	0.02
