# Supplementary material for: T vector velocity: A new ECG biomarker for identifying drug effects on cardiac ventricular repolarization
Source: PLoS One. 2019 Jul 8;14(7):e0204712. doi: 10.1371/journal.pone.0204712 (PMC6613676; doi:10.1371/journal.pone.0204712)

S6 Text. Distribution of QTcF, J-T_{peak}c and TrXc.

The graph below displays the cumulative distributions of QTcF, J-T_{peak}c and the T vector trajectory quantile parameters Tr10c to Tr100c from all ECGs in Study A and B from drug-free timepoints (placebo or pre-dose). N = 2395.

Note that the T vector trajectory quantiles are measured from 20 ms after the J point.

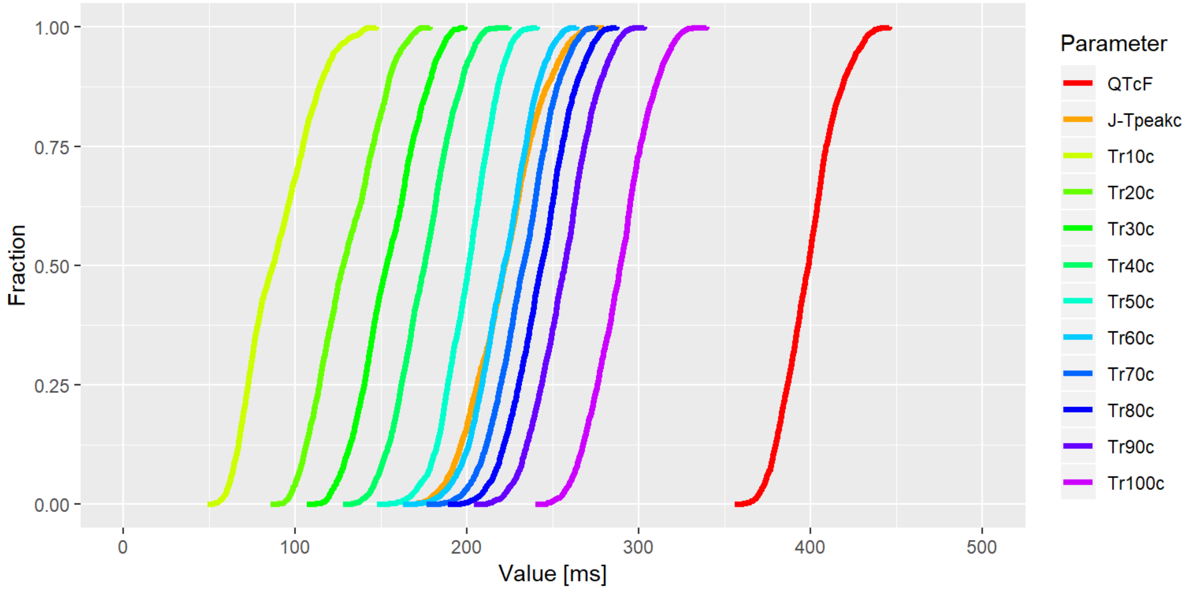

Supplement: S6 Text — (PDF) [file pone.0204712.s006.pdf]
